# “Left in limbo”: Exploring how patients with colorectal cancer interpret and respond to a suspected Lynch syndrome diagnosis

**DOI:** 10.1186/s13053-021-00201-1

**Published:** 2021-10-16

**Authors:** Nicole den Elzen, Sharelle L. Joseland, Sibel Saya, Sowmya Jonnagadla, Joanne Isbister, Ingrid Winship, Daniel D. Buchanan

**Affiliations:** 1 Colorectal Oncogenomics Group, Department of Clinical Pathology, Melbourne Medical School, The University of Melbourne, 305 Grattan Street, Parkville, Victoria 3010 Australia; 2 The University of Melbourne Centre for Cancer Research, Victorian Comprehensive Cancer Centre, Parkville, Victoria Australia; 3 Department of Paediatrics, The University of Melbourne, Parkville, Victoria Australia; 4 Department of General Practice, The University of Melbourne, Parkville, Victoria Australia; 5 Genomic Medicine and Family Cancer Clinic, The Royal Melbourne Hospital, Parkville, Victoria Australia; 6 Department of Medicine, The Royal Melbourne Hospital, The University of Melbourne, Parkville, Victoria Australia

**Keywords:** Suspected Lynch syndrome (SLS), Colorectal cancer (CRC), DNA mismatch repair (MMR) deficiency, Inconclusive diagnosis, Cancer risk management, Hereditary cancer

## Abstract

**Background:**

A diagnosis of suspected Lynch syndrome (SLS) is given when a tumour displays characteristics consistent with Lynch syndrome (LS), but no germline pathogenic variant is identified. This inconclusive diagnosis results in uncertainty around appropriate cancer risk management. This qualitative study explored how patients with CRC interpret and respond to an SLS diagnosis.

**Methods:**

Semi-structured telephone interviews were conducted with 15 patients with CRC who received an SLS diagnosis, recruited from cancer genetics services across Australia. Interviews were transcribed verbatim and analysed using thematic analysis. Participant responses were compared with appointment summary letters from cancer genetics services.

**Results:**

Participants’ interpretations of genetic test results were found to vary widely. While this variation often aligned with variation in interpretations by cancer genetics services, participants also had difficulties with the complexity and recall of genetic test results. Participants had a range of psychological responses to the uncertainty that their results presented, from relief to disappointment and doubt. Cancer risk perceptions also varied widely, with participants’ interpretations of their genetic test results just one of several influencing factors. Despite this variability, almost all participants adhered to cancer risk management advice, although different participants received different advice. All participants also communicated any cancer risk management advice to first-degree relatives, motivated by protecting them, but information communicated was not always consistent with advice received.

**Conclusions:**

Our study findings highlight the variability in patients’ interpretations of their diagnosis, cancer risk management and family communication when a diagnosis of SLS is received, and provide novel insights into how healthcare professionals can better support patients with SLS.

## Background

Lynch syndrome (LS) is a hereditary cancer predisposition syndrome caused by a germline pathogenic variant in one of four DNA mismatch repair (MMR) genes (*MLH1*, *MSH2*, *MSH6*, *PMS2*) or the *EPCAM* gene [1]. LS is the most common hereditary cause of colorectal cancer (CRC) and endometrial cancer [2–5], and also increases the lifetime risk of cancers of the ovary, stomach, small bowel, urinary tract, hepatobiliary tract, pancreas, prostate, brain and skin [6, 7].

Patients diagnosed with CRC are recommended to undergo screening for LS, which comprises tumour testing for the loss of expression (LOE) of one or more MMR proteins and/or high levels of microsatellite instability (collectively referred to as MMR-deficiency) [2, 4, 6–9]. If a colorectal tumour displays molecular features suggestive of LS, a diagnosis of LS is confirmed by the identification of a germline pathogenic variant in *MLH1*, *MSH2*, *MSH6*, *PMS2* or *EPCAM* [6, 9].

Importantly, in 50–60% of patients with colorectal tumours suggestive of LS, no germline pathogenic variant can be identified, resulting in a diagnosis of suspected Lynch syndrome (SLS) (also known as Lynch-like syndrome) [2, 4, 10]. Patients with SLS are likely a heterogeneous group, comprising both patients with undetermined sporadic and hereditary cancers [10, 11]. US and UK guidelines [12, 13] recommend that patients diagnosed with SLS be offered somatic genomic testing to identify the up to 70% of patients who have sporadic colorectal tumours with somatic inactivation of both copies of an MMR gene (referred to as “biallelic somatic MMR-deficiency”) [11, 14–18]. However, in Australia, this transition has been slow, resulting in suboptimal differentiation of patients with sporadic and hereditary MMR-deficient tumours [19], and uncertainty around their future cancer risks.

Currently, there are no clear Australian guidelines for managing the uncertain cancer risks of patients with SLS and their first-degree relatives, despite some recommendations that they be managed clinically as though they have LS [20]. This creates challenges for healthcare professionals around appropriate cancer risk management advice for these patients [10]. Australian cancer risk management guidelines for individuals with LS and their first-degree relatives are broadly consistent with international guidelines [6, 7, 9, 12, 13], and recommend 1–2 yearly colonoscopies and low-dose aspirin from 25 to 35 years; two-yearly gastroscopies and a subtotal colectomy may also be considered [21]. Females are also advised to have a hysterectomy and to consider a salpingo-oophorectomy [21].

An SLS diagnosis also creates challenges for genetic counsellors around effectively communicating uncertainty to patients, both regarding their inconclusive genetic test results and uncertain future cancer risks [22, 23]. This is likely to be exacerbated by the complexity of discordant tumour screening and genetic test results in an SLS diagnosis [24]. The majority of studies that have investigated patients’ cognitive, affective and behavioural responses to inconclusive genetic test results for a hereditary cancer syndrome have focussed on females undergoing genetic testing for hereditary breast and ovarian cancer [25]. These studies vary in the reported effects of inconclusive genetic test results on patients’ recall and comprehension of results, cancer risk perceptions, cancer-specific distress and worry, screening decisions and family communication [25, 26].

Despite the challenges around communicating and managing the uncertainty associated with an SLS diagnosis, no studies, to our knowledge, have examined cancer risk management advice provided to patients with SLS and their family members, and very few have explored how these patients interpret and respond to this information. Of the small number of studies that have examined patients’ interpretations and responses to an inconclusive LS diagnosis [24, 27, 28], all have been undertaken in North America and have included individuals with a variant of uncertain significance (VUS) in an MMR gene, which, while sharing similarities with an SLS diagnosis arising from an uninformative negative genetic test result, can differ substantially in genetic counselling approaches and patient responses [25, 29].

These studies have shown that even though many patients with an inconclusive diagnosis for LS misinterpreted or did not understand their genetic test results, most believed they had an increased risk of CRC and underwent regular cancer screening; around a third of female participants also had a hysterectomy and/or salpingo-oophorectomy to reduce cancer risks [27, 28]. Many participants also reported worrying about their or their families’ cancer risks, although this did not affect their mood in most cases [27, 28]. Katz et al. [27] also found that participants advised at least one family member of their CRC diagnosis and genetic test results. However, there were significant gaps in the communication of genetic test results to extended family members [27].

To ensure the best health outcomes for patients with SLS and their family members, a better understanding of their psychosocial and behavioural responses to an SLS diagnosis, and factors influencing these, is required. In this study, we used a qualitative approach to explore in-depth how patients with CRC experience an SLS diagnosis in the Australian health setting, including their interpretation of genetic test results, psychosocial impacts, cancer risk perceptions and management, and family communication. We also compared participants’ responses with diagnostic and cancer risk management advice provided by cancer genetics services to assess healthcare professional influence on patient understanding and behaviours.

## Methods

### Participant recruitment

The participants in this study were recruited from eligible individuals enrolled in the Applying Novel Genomic approaches to Early-onset and suspected Lynch Syndrome colorectal and endometrial cancers (ANGELS) study. The ANGELS study cohort includes individuals recruited from 16 cancer genetics services across six Australian states and territories.

ANGELS study participants were eligible for this study if they were: (a) 18 years or older; (b) diagnosed with an MMR-deficient colorectal tumour via MMR protein immunohistochemistry; (c) tested and no germline pathogenic variant was identified in the relevant MMR or *EPCAM* genes; and (d) for colorectal tumours displaying LOE of MLH1 and PMS2, a negative or inconclusive result was obtained for *MLH1* methylation and/or a *BRAF* p.V600E variant. Participants were excluded if: (a) their most recent primary cancer diagnosis was not CRC; (b) a germline VUS in an MMR or *EPCAM* gene was identified; (c) they were receiving palliative care; or (d) they were non-English-speaking. Purposive sampling based on gender, location, age of CRC diagnosis, cancer stage and tumour MMR protein expression was used to select an information-rich sample.

### Data collection and analysis

A semi-structured interview guide, previously reviewed by several individuals living with cancer, was piloted with research team members and feedback incorporated. All participant interviews were conducted via phone by NDE, audio-recorded and transcribed verbatim. Participants were asked about their understanding of genetic test results, psychosocial impacts, perceptions of personal and family cancer risks, cancer risk management advice and behaviours, and family communication of genetic test results and cancer risk management advice.

Thematic analysis based on inductive coding [30] was undertaken by NDE with the assistance of NVivo12 (QSR International Pty Ltd., Melbourne Australia). A reflexive approach was used, where codes evolved during analysis and were clustered to generate themes of shared meaning grounded in the data [31]. A sample of transcripts was independently coded by research team members (SJ, SS and JI) to achieve analytical rigour. Recruitment was completed when no new significant themes emerged, suggesting thematic saturation [32].

Participant interpretations of genetic test results and recall of cancer risk management advice were compared with information included in appointment summary letters from the relevant cancer genetics service to the participant and/or their doctor(s). Original letters or relevant excerpts were provided for 14 of 15 participants, and a summary of cancer risk management advice was provided for one other participant.

Participant demographic, medical and familial information was retrieved from patient records obtained through the ANGELS study, and supplemented with self-reported information from participant interviews.

### Ethics approval

This study and the ANGELS study were approved by The University of Melbourne Human Research Ethics Committee (Ethics IDs 1955697 and 1750748).

## Results

### Participants

Of 21 eligible individuals approached, 15 (71%) consented to participate. Participant characteristics are summarised in Table 1. Interviews occurred between March and July 2020 and ranged from 30 to 110 min in duration (mean: 65 min). The average period between participants’ genetic diagnosis and study interview was 2.2 years (range: 0.5–3.8 years).

**Table 1:** Participant characteristics (*n* = 15)

Characteristic	Categories	n (%)
Gender	Male	8 (53)
	Female	7 (47)
Age at CRC diagnosis^a^	25–34	3 (20)
	35–44	6 (40)
	45–54	2 (13)
	55–64	1 (7)
	65 or older	3 (20)
Education level	High school	2 (13)
	Professional certificate/diploma	4 (27)
	Bachelor degree and above	9 (60)
Ethnicity	Caucasian	12 (80)
	Non-Caucasian	3 (20)
Occupation^b^	Non-health-related	9 (60)
	Health-related	6 (40)
Pathological stage of CRC^c^	Stage I	6 (40)
	Stage II	7 (47)
	Stage III	2 (13)
	Stage IV	0 (0)
Tumour MMR immunohistochemistry	LOE MLH1 and PMS2	7 (47)
	LOE MSH2 and MSH6	4 (27)
	LOE MSH6	2 (13)
	LOE MSH6 and PMS2	1 (7)
	LOE PMS2	1 (7)
Number of LS-associated primary cancers^d^	1	14 (93)
	2	1 (7)
Number of first- and second-degree relatives with LS-associated cancers^d^	0	7 (47)
	1	4 (27)
	≥2	4 (27)
Australian state/territory	Victoria	8 (53)
	New South Wales	2 (13)
	Australian Capital Territory	2 (13)
	Western Australia	2 (13)
	Queensland	1 (7)
Geographic location	Metropolitan	10 (67)
	Regional	5 (33)

^a^ For the one participant with multiple LS-associated cancers, age at their earliest cancer diagnosis was used

^b^ Health-related occupations included a clinician, medical imager, medical receptionist, medical researcher, nurse and psychologist. Non-health-related occupations included the areas of teaching, information technology, aviation, meteorology, hairdressing/beauty therapy, trades, project management and administration

^c^ Cancer stage was based on the American Joint Committee on Cancer (AJCC) tumour-node-metastasis (TNM) system (7th or 8th edition) [33, 34]

^d^ LS-associated cancers include CRC, endometrial carcinoma, small bowel adenocarcinoma and ureter or renal pelvis cancer as per Amsterdam II criteria [35]. Cases of kidney cancer where further details of the type of kidney cancer were not available were included

### Themes

Five key themes emerged from participant interviews: genetic diagnosis was unclear; the double-edged sword of an uncertain genetic diagnosis; assimilating genetic test results with other beliefs and evidence; healthcare professional advice determined cancer risk management behaviours; communication protects family members.

Themes are illustrated by representative quotes of participants, identified by pseudonyms. For ease of reading, quotes exclude disfluent speech and have been shortened in some cases (indicated by “…” ) without affecting participant meanings.

### Genetic diagnosis was unclear

#### Variable healthcare professional interpretations of LS testing results

Participants’ interpretations of genetic test results varied widely (Table 2), including five participants misinterpreting their results to mean there was no possibility they had LS or a hereditary cancer predisposition.

**Table 2:** Representative quotes displaying variable participant interpretations of genetic test results

Interpretation	Representative quote
Do not know	“To be honest, I don’t remember much of it. And I felt like I did not get the exact answer, if it was passed on to me or was it environmental. Until today, I’m not sure.” – *Donna*
Do not have LS	“… she had talked to me about, well the result is that there’s no mutations found in those specific genes. I think there were five or six genes which were involved in that. And it means that you haven’t got Lynch syndrome … So, the chances are that this was just one of those environmental or acquired tumours for unknown reasons.” – *Daniel* “I was just told that I didn’t have Lynch syndrome, and that it wasn’t a genetic cancer.” *– Jodie*
Inconclusive, but unlikely LS	“… he said I’m in this small group, or this group of patients for whom it’s not a definitive link but there’s a chance, and they can’t rule it out, but it’s unlikely that it is a genetic issue.” *– Matthew*
Inconclusive for LS	“I think it was basically inconclusive. I don’t know. I still really don’t even really remember. But it wasn’t of a concern.” *– Trevor* “Well, it just seemed she didn’t know whether I had it or I didn’t have it.” *– Brian* “So the advice at that stage was that everything’s negative, but the suspicion’s still there.” – *Peter*
Inconclusive, but likely LS or treat as LS	“… the woman we spoke to sort of said, oh well, that’s nothing to worry about, and nothing you can do about that … and then I got another letter saying, oh, well actually because it was sort of indeterminate, or sort of an undetermined result, that they actually thought I would be better off being treated like I had a positive result.” – *Veronica* “… from their experience, they told me that it most likely is [LS], but the technology hasn’t caught up to actually identify it for certainty.” *– Colin*

Healthcare professional advice was a likely source of some of this variability, with some appointment summary letters from cancer genetic services indicating a reduced suspicion or unlikely chance of LS, and others that LS was either possible or likely. In some cases, a participant’s personal or family history of LS-associated cancers (or lack thereof), young age of cancer diagnosis and/or other relevant information was provided as an explanation for the clinician’s assessment. No letters ruled out the possibility of LS or a hereditary cancer predisposition, illustrating that participant interpretations did not always align with healthcare professional advice received.

#### Complex and conflicting test results

For some participants, multiple lines of testing and conflicting immunohistochemistry and genetic test results also affected the clarity of their genetic diagnosis.

“… they also re-checked the tumour … to see about the Lynches syndrome in it. And it was found that, yes, it most definitely did have. The blood tests when they came back showed me as negative, I didn’t have, which is a very strange thing to me …” – *Hugh*

“… the first level of testing showed up something wasn’t right. And then the next test they did was negative, and it said, no it wasn’t. So then they did another level, and that was sort of undecided. So in the end, they really sort of said, well, we’re not really sure.” – *Veronica*

#### Difficulty recalling genetic information

Around half of participants also had difficulty recalling genetic information, raising their transitory interaction with cancer genetics services, the high volume of other medical appointments, receiving diagnostic information from multiple healthcare professionals, or the effects of their illness or treatment on cognition.

“… a lot of that time’s a bit hazy in my memory … It was a bit of a swirl at the time of information and just go here, go there, get blood tests, see this person.” – *Trevor*

“… I probably didn’t pay much attention … I haven’t had a huge amount to do with [the cancer genetics service].” – *Ian*

### The double-edged sword of an uncertain genetic diagnosis

Despite differences in the interpretation of genetic test results, there was a common sense of uncertainty among participants about their genetic diagnosis, and variable psychological responses to this.

#### No explanation for my cancer

Participants who interpreted their results as meaning they did not have LS, often expressed relief, but also sometimes disappointment or doubt because they did not have an explanation for their cancer.

“I felt relieved actually … It would have been quite confronting to have to face the hysterectomy or anything like that.” – *Jodie*

“You sort of think, oh well, I guess if there’s no genetic issue, then I guess my kids are not going to be affected by it; but it still doesn’t explain why it happened to me, kind of thing. Are my kids still going to be at risk? Or is it just a once off variation … ?” – *Victoria*

#### No definitive result

Similarly, participants who interpreted their results as inconclusive for LS sometimes expressed relief that the result was not positive, but also disappointment or lingering doubt due to the uncertainty of their result.

“I guess in some ways it was maybe a relief … But then, just because it wasn’t detected, doesn’t mean it’s not there. So then I’m sort of left in limbo about what the risk actually is and if it is a risk of being passed on.” – *Peter*

#### Acceptance of uncertainty

Other participants accepted the uncertainty associated with their diagnosis, in some cases acknowledging that their genetics or cancer was out of their control.

“… it is what it is. I can’t change my genetics … so I’ll deal with the consequences as they come along.” – *Ian*

“You have to let go of that why, why, why, why. Why did this happen? … It’s just been a bit of a life lesson to not have an answer for everything.” – *Jodie*

#### Resigned to fate

Several participants who interpreted their results to mean it was likely they had LS appeared resigned to dying from cancer.

“… I always figured the diabetes would take me out in some form, not cancer. And so, it was just like, oh well, you know, this is the way.” – *Veronica*

However, these participants also discussed the positive impact of their genetic diagnosis on their life and outlook.

“… things that I’d kind of thought, I’m too busy to do that, I’ll do it another time, now I seem to sort of think, no, let’s do it now. And I think I sort of actually seek out things that I enjoy more now. That’s just because you just don’t know.” – *Veronica*

### Assimilating genetic test results with other beliefs and evidence

#### Cause of cancer

When asked what they believed caused their cancer, some participants felt that they did not know, while others believed it was either bad luck, due to lifestyle or environmental risk factors, or likely hereditary. In forming their beliefs, participants’ genetic test results were often just one of several considerations, along with their age of cancer onset, multiple cancer diagnoses, family history of cancer, and/or environmental or lifestyle factors.

“I think it’s genetics. There’s a history of cancers in my dad’s family. I don’t think it’s lifestyle factors. I know my younger sister had uterine cancer … and she had a really healthy lifestyle.” – *Rachel*

“I just think it’s bad luck … I don’t have any family history. I have always lived a very healthy lifestyle … So all I can put it down to is bad luck.” – *Jodie*

#### Cancer risk perceptions

Most participants understood that LS increases certain cancer risks. However, consistent with different beliefs about the likely cause of their cancer, participants’ future cancer risk perceptions also varied widely. In forming perceptions of their future cancer risks, participants assimilated their genetic test result with other beliefs and evidence of cancer risks, including their CRC diagnosis, young age of cancer onset, multiple cancer diagnoses, life stage, regular cancer screening, surgical removal of organs, and/or lifestyle and environmental risk factors (Table 3).

**Table 3:** Representative quotes displaying participant variability in cancer risk perceptions and influencing factors

Cancer risk perception	Representative quotes illustrating factors influencing perceptions
Do not know cancer risk	“… it was inconclusive, so you wouldn’t know. I don’t know whether that’s good or bad*.*” *– Brian* “… there’s the risk of developing a Lynch-like or well Lynch-associated malignancy, which is difficult to define seeing as I don’t have a firm diagnosis of Lynch.” – *Peter*
Low cancer risk	“Well, another cancer of the same type, I think is pretty much zero. But any other cancer, I dunno … I’m just sort of fairly confident based on nothing really that I won’t get any other type of cancer.” *– Trevor* “He said because you’ll be so heavily screened for any cancers, you have a lower risk than the rest of the population.” *– Jodie* “I’ve got very low risk factors. I’ve never smoked. I’m pretty fit and active. I eat a balanced diet. There are no obvious signs … if I do look after myself, then there’s no reason why I would see myself in a situation where I’m having a recurrence.” – *Matthew*
Population level cancer risk	“… I just find probably it’s the same risk as anybody else*.” – Amy*
Increased cancer risk	“… you probably tend to think, oh, now I’ve had that cancer, maybe I’ll get another.” – *Amy* “… because I got the bowel cancer early, there’s a good chance that I will get another tumour, because my bowel is obviously already of the type that will get tumours … and it’s purely probably that genetic thing.” – *Veronica* “… while I was doing the radiation, she got me to fill something in that said there’s a risk from developing other things from this radiation … and because I’m doing a lot of tests and like CTs and all that, that increases my chances.” *– Donna*

### Healthcare professional advice determined cancer risk management behaviours

#### Variable healthcare professional advice

Participants discussed receiving cancer risk management advice from their colorectal surgeon, oncologist and/or cancer genetics service. All but one participant said they were advised to have regular colonoscopies (ranging from annually to three-yearly). However, only four participants said they were advised to take aspirin, and only around half of female participants mentioned advice to consider a hysterectomy and/or salpingo-oophorectomy.

An analysis of appointment summary letters from cancer genetics services confirmed variability in cancer risk management advice provided to participants, and strong concordance of participant responses with advice received. Of 15 letters, seven included patient cancer risk management advice. Of these, all but one recommended regular colonoscopies, ranging from annually to two-yearly. Only two letters recommended low-dose aspirin, and one recommended following LS cancer risk management guidelines, which include a recommendation to take low-dose aspirin [21]. None of the letters to seven female participants discussed consideration of a hysterectomy or salpingo-oophorectomy, although two females had already had these procedures. All participants who received advice to undergo colonoscopies and/or take low-dose aspirin in their appointment summary letter recalled this advice, including the recommended frequency of colonoscopies.

#### Adherence because it was recommended

All participants said they adhered to cancer screening advice, and all but one participant indicated that they adhered to any advice received regarding risk-reducing surgery and aspirin.

Many participants said they followed cancer risk management advice because it was recommended.

“I don’t understand completely why they’ve said two years [for colonoscopies], but if that’s what their recommendation is, well then that’s what the recommendation is. So I’ll go with it.” *– Amy*

#### Adherence protects me from cancer

Others discussed how cancer screening or risk-reducing surgery decreased cancer risk, caught cancer early, made them feel protected and reduced cancer anxiety.

“I feel very protected by the process of the fact that somebody every year is going to check me.” – *Jodie*

“With three-yearly scans, I just will have more opportunities for something to be noticed should something come up.” – *Matthew*

#### Barriers were insufficient to prevent compliance

Around half of participants raised barriers to cancer screening, including the unpleasant nature of colonoscopies, risks associated with procedures and access issues. However, these were insufficient to prevent adherence to advice.

“I always get really nervous as well when you go under anaesthetic … But I’m happy to do those things because they’re recommended.” – *Anna*

### Communication protects family members

#### Variable family cancer risk management advice

Those participants who recalled receiving family cancer risk management advice indicated that they communicated this advice to relevant family members. However, a comparison of participants’ recall of advice with the advice contained in appointment summary letters showed inconsistencies in 10 out of 13 cases. Inconsistencies included: recalling advice for a one-off (rather than ongoing) colonoscopy; recalling more frequent colonoscopies than advised; not recalling the consideration of low-dose aspirin or a hysterectomy and/or salpingo-oophorectomy; and not recalling all relevant first-degree relatives for whom advice was provided.

There was also variability in family cancer risk management advice provided in appointment summary letters. The recommended frequency of colonoscopies ranged from five-yearly to 1–2 yearly. One letter recommended a single baseline colonoscopy, while another recommended faecal occult blood tests (FOBT) from age 40, followed by 5-yearly colonoscopies from ages 50–74. Only two letters recommended consideration of low-dose aspirin, and one raised the consideration of a hysterectomy and/or salpingo-oophorectomy in females. Two cancer genetics service letters did not provide family cancer risk management advice.

#### Communicating to protect family members

All but two participants said they also communicated their genetic test results to their first-degree relatives (excluding young children). Communication of genetic test results to extended family members, however, was less consistent.

A common motivation for communicating to family members was to protect them. Participants who interpreted their results as inconclusive for LS were motivated to protect their family from the risk of cancer. Participants who interpreted their results as meaning they did not have LS, communicated this to protect family members from worry.

“I think that the family should know … At least warn them because cancer, you can’t cure cancer; you can only just prevent it and get it at a early stage.” – *Colin*

“My mum told her sisters and brothers who could possibly have been impacted if it was a genetically caused cancer … it would have been a heightened sense of worry for them.” – *Jodie*

#### Non-disclosure to protect family members

Paradoxically, protecting family members from worry was also a reason for not communicating genetic results, with the uncertainty of results or cancer cause perceived as a source of unnecessary worry (Table 4).

**Table 4:** Representative quotes of barriers to family communication of genetic test results

Communication barriers	Representative quotes
Protect from worry	“I don’t want to scare them yet. I want to know the answers myself first before I let them know. So I haven’t discussed anything.” *– Donna* “I think they’re all in another state and … I’ve got a cousin with a brain cancer. It just seemed too close to home at the time to mention it.” – *Rachel*
Insignificance of uncertain result	“… the genetic test results didn’t really seem significant enough for sort of more wide-spread communication.” – *Matthew*
Difficulty passing on bad news	“I was actually quite sort of anxious about telling them that … only because I guess I felt like I was handing them on potentially bad news for their health.” – *Veronica*
Lack of relevance to family member	“He’s got no kids and he’s in America at the moment. We will contact him if something came up, but he’s older than Mum. He’s in his 70’s. So I think it would be little relevance to him.” – *Peter*
Distance / No contact	“For my other aunts because I don’t have their contacts or I’m not too close with them, I just can’t tell ‘em.” – *Colin*
Family resistance	“I don’t think they all agreed with that … or believing that there was some sort of genetic issue in the family. They probably got a little bit defensive actually, now that I think about it …” – *Anna*
Cultural barriers / Taboo subject	“Because in Chinese this is like a taboo topic. You don’t talk about this. It’s a stain on the family.” – *Colin*

#### Multiple barriers to communicating to extended family

Other barriers to communicating genetic test results to extended family members included the perceived insignificance or unhelpfulness of an uncertain result, perceived lack of relevance to the family member (due to old age, no children or no history of cancer in that branch of the family), cultural barriers, difficulty passing on bad news or discussing a taboo subject, family resistance to news, and distance or lack of contact (Table 4).

## Discussion

This study explored how patients with CRC interpret and respond to a diagnosis of SLS. It revealed variability in patients’ interpretation of genetic test results, cancer risk perceptions, cancer risk management behaviours and advice communicated to family members. It also provided novel insights into factors influencing patients’ interpretation of and responses to an SLS diagnosis, particularly the key role of cancer genetics services and healthcare professionals.

### Interpretation of SLS diagnosis

Genetic information is inherently complex, and previous studies have shown that patients can have difficulty understanding and recalling genetic information discussed at the time of their genetic diagnosis [36–38]. In the case of an SLS diagnosis, this complexity is exacerbated by the complexity and ambiguity of a tumour screening result suggestive of LS, followed by an uninformative negative genetic test result for LS [24]. According to Mishel’s Theory of Uncertainty in Illness [39], ambiguity, complexity and unpredictability of illness gives rise to uncertainty, which can lead to a diverse range of patient cognitive, emotional and behavioural responses.

In concordance with this and previous studies [24, 27], we found that patients with CRC who received a diagnosis of SLS varied widely in their interpretations of their genetic test results, including some misinterpreting their results to mean they did not have a potentially heritable component to their CRC. Previous studies also reported that some patients with an SLS diagnosis interpreted their results as positive for LS [24, 27], which was not a finding here, perhaps due to differences between the cohorts and health systems of these studies. Further, we showed that some patients diagnosed with SLS experienced uncertainty or confusion from the complexity of multiple lines of tumour and germline testing and the ambiguity of discordant tumour and genetic test results. Similar patient confusion from multiple lines of tumour and germline testing has previously been reported [24, 40]. Around half of participants also had difficulty recalling genetic information, raising the impacts of cancer treatment or many medical appointments on their comprehension and recall of genetic test results, again consistent with previous reports [41–43]. The brevity of interactions with cancer genetics services was also raised by some participants as affecting information recall.

Because patients can have difficulty comprehending and recalling information discussed at genetic counselling appointments, genetics services often provide an appointment summary letter to patients to reinforce information discussed and to facilitate the communication of genetic information and advice to family members or healthcare professionals [44]. Importantly, an analysis of participants’ appointment summary letters in this study showed that some of the variability in participants’ interpretations of their genetic test results was likely to have arisen from variability in guidance from cancer genetics services. This variability in advice was due in part to efforts by some cancer genetics services to reduce the uncertainty associated with an SLS diagnosis, using the age of cancer diagnosis and a personal or family history of LS-associated cancers to assess the likelihood of LS in their patients. However, this information cannot reliably differentiate patients with LS from those with biallelic somatic MMR-deficiency [16, 17, 45]. In the absence of the necessary tumour genomic testing to provide a definitive diagnosis, this uncertainty and variability in the interpretation of SLS diagnoses will continue.

### Psychological response

Participants’ affective responses to their genetic test results ranged from relief through to disappointment or lingering doubt, irrespective of their interpretations of results. Feelings of relief stemmed from the lack of a definitive positive result for LS. Disappointment and doubt were associated with the uncertainty of an inconclusive result or the lack of an explanation for the participant’s cancer.

A wide spectrum of affective responses has previously been reported for patients who received inconclusive genetic test results for a hereditary cancer syndrome [24, 25]. According to Mishel’s Theory of Uncertainty in Illness [39], patients’ experiences of uncertainty depend on whether they perceive the uncertainty as an opportunity, danger or both. This appeared to be the case in this study, where psychological responses were discussed in the context of participants’ and family members’ future cancer risks and/or the need for a hysterectomy and salpingo-oophorectomy. The acceptance of uncertainty by many participants might reflect their previous experience with uncertainty from their CRC diagnosis or their perceived ability to control this risk through cancer screening [26].

### Cancer risk perceptions

Participants’ beliefs about the cause of their cancer and their future cancer risks also varied, with genetic test results just one of several influencing factors. This is consistent with an extensive literature showing that a complex range of factors influence cancer risk perceptions among individuals with an increased risk of hereditary cancer [46]. Studies of individuals with LS have also found variable perceptions of cancer risks [47, 48]. Further, Katz et al. [27] reported that 71% of participants with CRC and an inconclusive diagnosis for LS believed that their future CRC risk was no higher than that of “other CRC survivors”. This might be explained by the findings here that only some participants believed that their cancer was likely hereditary, and that cancer risks perceptions incorporated reductions in risk from regular cancer screening, risk-reducing surgery and positive lifestyle behaviours.

### Cancer risk management behaviours

Almost all participants reported adhering to all cancer risk management advice, irrespective of their interpretation of their genetic test results, perceived cancer risk, or any barriers to cancer screening. This finding augments the finding of Katz et al. [27] that patients with CRC who received an inconclusive diagnosis for LS underwent regular cancer screening. It also aligns with findings that cancer risk perceptions do not predict screening behaviours in individuals with LS [49], whereas a personal history of CRC does [50]. Further, in studies of patients diagnosed with breast cancer, the decision to undergo cancer screening was found to be based on participants’ cancer diagnosis prior to genetic testing [51–53]. This might explain why the patients with CRC in this study adhered to cancer risk management advice, even if they misinterpreted their results to mean there was no possibility they had LS or a hereditary cancer predisposition.

Importantly, cancer risk management advice provided to participants by cancer genetics services varied. While cancer genetics services are only one of several potential sources of cancer risk management advice for patients with SLS, participants’ cancer risk management behaviours did appear to align with this advice. To the best of our knowledge, this is the first time a study has explored cancer risk management advice provided to cancer patients with SLS. The variability in cancer risk management advice is perhaps unsurprising given uncertainty around cancer risks in this heterogenous cohort of patients, together with the lack of clear Australian guidelines for cancer risk management in patients with SLS.

### Family communication

It is important that patients with SLS communicate cancer risk management advice to first-degree relatives to help manage family members’ elevated cancer risks [54–56]. Consistent with previous findings [27], we found that all participants who received family cancer screening advice communicated this to family members. However, we also showed that participants’ recall of advice often did not align with advice received from cancer genetics services, which itself was variable. Participants’ difficulty recalling correct cancer risk management advice for family members aligns with many participants’ difficulty recalling genetic information in general, and may be attributed in part to the complexity and uncertainty of their genetic diagnosis [24, 25]. Also, those participants who misinterpreted their results as meaning there was no possibility they had LS or a hereditary cancer predisposition, communicated this information to family members. Together, these factors are likely to contribute to the suboptimal cancer screening reported for relatives of CRC patients diagnosed with SLS [57].

Consistent with findings for other patients diagnosed with cancer [58], the principal motivation for communicating genetic test results and screening advice to family members was to protect them. Barriers to family communication also aligned with those previously reported for individuals diagnosed with cancer or LS, including avoiding family worry, perceived irrelevance of results to the family member, cultural barriers, difficulty discussing a taboo subject or passing on bad news, family resistance, and demographic or emotional distance from relatives [58–61]. The perceived insignificance or unhelpfulness of uncertain results was an additional barrier to broader family communication of an SLS diagnosis, consistent with findings for patients diagnosed with breast cancer who received uninformative genetic test results [62].

### Study strengths and limitations

This study is the first to explore patient experiences of an SLS diagnosis in the Australian health setting. The qualitative methodology, together with the analysis of cancer genetics service appointment summary letters, enabled insight into the complexity of patients’ understanding, perceptions and behaviours and factors influencing these. Further, through purposive sampling and multi-site recruitment, we were able to capture diversity in participant demographics and experiences. However, individuals of varied cultural backgrounds were under-represented in this study, and tertiary-educated individuals were over-represented. Thus, care should be taken in generalising findings. All participants were also interviewed within 4 years of receiving their genetic test results; thus, cancer risk management behaviours do not necessarily reflect long-term behaviours.

### Practice implications and recommendations

This study reaffirms the critical role of cancer genetics services in facilitating patients’ understanding of an SLS diagnosis and managing their and their families’ future cancer risks. Findings highlight the need for clear, consistent information to minimise the misinterpretation by patients of discordant tumour and germline test results. Genetic counselling could also address the potential for patients to perceive an SLS diagnosis as insignificant or worrying for family members. It is warranted for cancer genetics services to contact patients 1–2 years post-testing to facilitate patient recall of genetic information and family cancer risk management advice.

Finally, this study has highlighted the importance of Australian cancer risk management guidelines for patients receiving an SLS diagnosis and their first-degree relatives to enable more consistent cancer risk management advice based on optimal diagnostic information. As in the UK and US [12, 13], we suggest that these guidelines recommend genomic testing of colorectal tumours of patients diagnosed with SLS to identify a significant proportion of those with sporadic cancers related to biallelic somatic MMR-deficiency, and provide appropriate, evidence-based cancer risk management advice for the remaining patients and their first-degree relatives.

## Conclusions

Patients with SLS are a heterogeneous group who pose challenges for healthcare professionals around communicating an inconclusive genetic diagnosis and managing uncertain cancer risks. Our study demonstrated variability in patients’ interpretations of their SLS diagnosis, cancer risk management and family communication. It also highlighted the key role of cancer genetics services in influencing patients’ responses, and showed that variable healthcare professional advice was a source of variable patient interpretations of their genetic diagnosis and cancer risk management behaviours. Further research is needed to explore cancer risk management advice provided to patients with SLS by other treating healthcare professionals, and factors influencing cancer risk management behaviours in first-degree relatives.

## Data Availability

The datasets generated and/or analysed during the current study are available from the corresponding author on reasonable request.

## Abbreviations

AJCC: American Joint Committee on Cancer
ANGELS study: Applying Novel Genomic approaches to Early-onset and suspected Lynch Syndrome colorectal and endometrial cancers study
CRC: Colorectal cancer
LOE: Loss of expression
LS: Lynch syndrome
MMR: DNA mismatch repair
SLS: Suspected Lynch syndrome
TNM: Tumour-node-metastasis
VUS: Variant of uncertain significance
